# Is synaesthesia more common in autism?

**DOI:** 10.1186/2040-2392-4-40

**Published:** 2013-11-20

**Authors:** Simon Baron-Cohen, Donielle Johnson, Julian Asher, Sally Wheelwright, Simon E Fisher, Peter K Gregersen, Carrie Allison

**Affiliations:** 1 Autism Research Centre, Department of Psychiatry, University of Cambridge, Douglas House, 18B Trumpington Rd, Cambridge CB2 8AH, UK; 2 Max Planck Institute for Psycholinguistics, 6500 AH, Nijmegen, The Netherlands; 3 Donders Institute for Brain, Cognition and Behaviour, Radboud University Nijmegen, Nijmegen, The Netherlands; 4 Robert S. Boas Center for Genomics and Human Genetics, The Feinstein Institute for Medical Research, North Shore-LIJ, Manhasset 11030, NY, USA

## Abstract

**Background:**

Synaesthesia is a neurodevelopmental condition in which a sensation in one modality triggers a perception in a second modality. Autism (shorthand for Autism Spectrum Conditions) is a neurodevelopmental condition involving social-communication disability alongside resistance to change and unusually narrow interests or activities. Whilst on the surface they appear distinct, they have been suggested to share common atypical neural connectivity.

**Methods:**

In the present study, we carried out the first prevalence study of synaesthesia in autism to formally test whether these conditions are independent. After exclusions, 164 adults with autism and 97 controls completed a synaesthesia questionnaire, Autism Spectrum Quotient, and Test of Genuineness-Revised (ToG-R) online.

**Results:**

The rate of synaesthesia in adults with autism was 18.9% (31 out of 164), almost three times greater than in controls (7.22%, 7 out of 97, *P* <0.05). ToG-R proved unsuitable for synaesthetes with autism.

**Conclusions:**

The significant increase in synaesthesia prevalence in autism suggests that the two conditions may share some common underlying mechanisms. Future research is needed to develop more feasible validation methods of synaesthesia in autism.

## Background

Synaesthesia occurs in 4% of the population [1]. Autism spectrum conditions (henceforth, autism) occur in 1% of the population [2]. If these conditions are independent, then synaesthesia and autism should co-occur in only 4 in 10,000 people. However, both are thought to involve atypical neural connectivity [3-5], which may point to a shared aetiology.

Synaesthesia occurs when the stimulation of one sensory modality automatically evokes a perception in another unstimulated modality [6]. The most common forms of synaesthesia involve written and/or auditory stimuli triggering colours [1,7]. Individuals with ‘developmental synaesthesia’ report having the condition for as long as they can remember [8]. Developmental synaesthesia shows familial clustering and (as yet unidentified) genetic factors are likely to play a significant role [9-11]. Developmental synaesthesia is distinct from ‘acquired synaesthesia’ where individuals report first experiencing synaesthesia later in life, after an inducing event such as the use of hallucinogenic drugs [12]. Developmental synaesthesia is not easily explained by learning because siblings with this condition, exposed to similar environments, often report different colours for the same inducer, or experience different variants of the trait [10], although this has been disputed [13]. Crucially, people with developmental synaesthesia describe their experiences as automatic and involuntary, in contrast to metaphoric associations that are voluntary and are not intended to be taken literally [14].

Neuroimaging studies confirm that synaesthesia is associated with differences in brain structure and/or function [15-18]. The hyper-connectivity hypothesis proposes that people with synaesthesia have excessive neural connections between different regions, connections that are diminished or absent from unaffected individuals [5,19-21]. Evidence for this hypothesis comes from a diffusion tensor imaging study [18] showing that people with grapheme-colour synaesthesia have increased white matter connectivity compared to unaffected controls.

Autism (including Asperger syndrome) involves social and communication difficulties, alongside unusually narrow interests and activities and resistance to change [22]. Neuroimaging studies of autism indicate that the autistic brain is anatomically and functionally different from the typical brain [3,23,24]. Differences are found in grey and white matter, and cortical connectivity [25,26]. According to one hypothesis, autism is associated with a reduction in long-range neural connections, alongside an increase in local, short-range connectivity [3,4]. This hypothesis may explain aspects of autism such as detail-oriented processing (‘obsessions’). Thus, an increase in local connectivity may play a role in both autism and synaesthesia.

Ten percent of individuals with autism have savant skills (a skill that is above average for the general population), and an estimated 50% of savants have autism [27]. Daniel Tammet, who has both Asperger syndrome and synaesthesia, and who is a memory savant (he memorized Pi to 22,514 decimal places) inspired the hypothesis that savantism arises in individuals who have both autism and synaesthesia. This combination of conditions has been speculated to give rise to strong ‘systemizing’ and excellent attention to detail, both products of neural hyper-connectivity [8,28].

Beyond such single-case reports of synaesthesia in autism, it has been proposed that synaesthesia may be common in autism [29,30]. The idea has tentative indirect support from two different lines of investigation. First, a molecular genetic study of families with auditory-visual synaesthesia found linkage to an area on chromosome 2, in a region that had previously been linked to autism [9]. However, these genetic findings may not overlap since the study by Asher et al. found considerable genetic heterogeneity across families. Moreover, *cAMP-GEFII*, an interesting candidate gene from the linkage region, harbours rare variants that are associated with autism but not implicated in synaesthesia. Secondly, an event-related potential study found that participants with autism showed occipital (visual cortical) activity while attending to auditory stimuli [31], although the interpretation of this anomaly is unclear. In the present study, we carried out the first direct assessment of overlap between the two conditions.

## Methods

In total, 172 adults with autism and 123 typical adults responded and gave electronic consent. These are the subset of those who responded from an email sent to 927 adults with autism and 1,364 typical adults. Participants (aged 18 years old or older) were invited to visit one of two websites (http://www.autismresearchcentre.com or http://www.cambridgepsychology.com) hosted by the Autism Research Centre at Cambridge University. Potential participants received an email inviting them to participate in a study on synaesthesia in adults with and without autism. The email defined and briefly described synaesthesia; the definition of synaesthesia^a^ was provided in the informed consent form. In order to reduce sampling bias, the consent form stressed that all individuals – with autism or synaesthesia, both, or neither – were eligible to participate.

The study was approved by the Psychology Research Ethics Committee of the University of Cambridge. All participants with autism had a diagnosis from a clinical psychologist or psychiatrist from a recognized clinic. All participants were asked to complete two questionnaires online:

**Figure 1:**
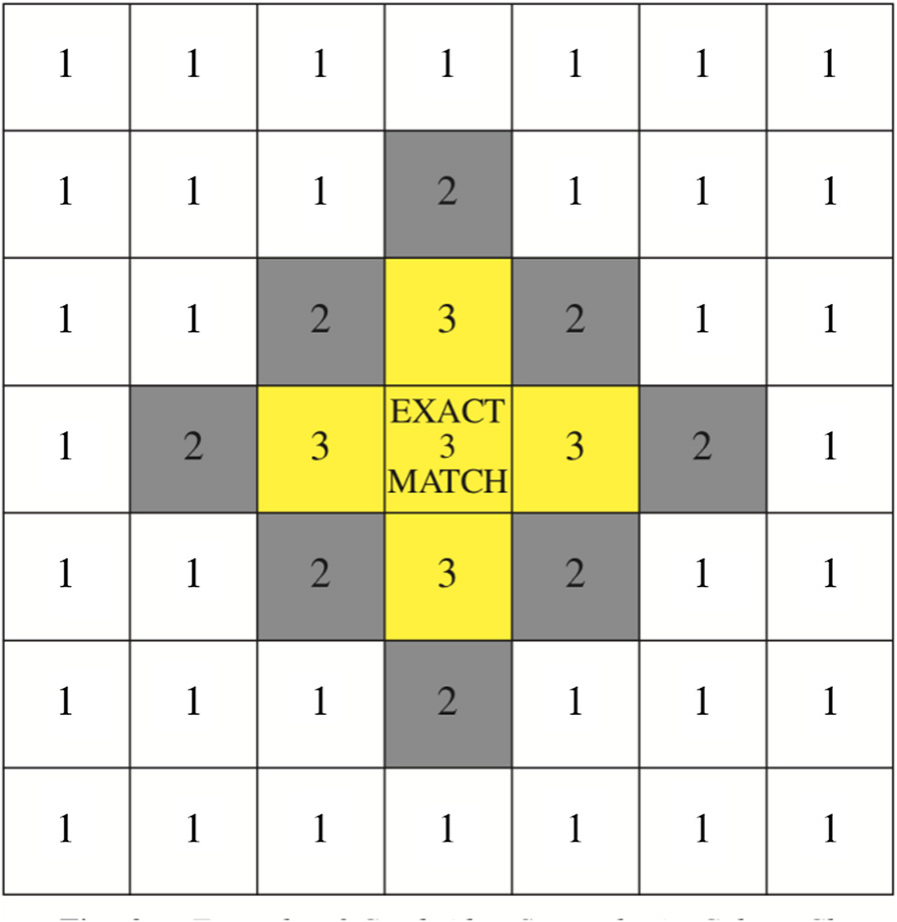
**Scoring protocol for the ToG-R.** Each box represents a swatch on the colour chart. From Asher et al. (2006) [32].

(a) The Synaesthesia Questionnaire*,* adapted from previous studies [9,32]. This was used as a further form of self-report of synaesthesia and to document the subtype(s) experienced. In order to screen out acquired synaesthesia, it also asked a series of questions used as exclusion criteria: whether the person had any medical conditions affecting vision (e.g., colour blindness), any medical condition affecting the brain (e.g., head injury, epilepsy, brain tumour, or stroke), any history of hallucinogenic drug use, and how long the person had experienced synaesthesia. Conservative inclusion criteria were used to judge if any individual had synaesthesia. If any of these questions were answered positively, or if synaesthesia was first experienced in adulthood, then the person was conservatively judged not to have synaesthesia.

(b) The Autism Spectrum Quotient (AQ) [33]. This was used to measure the number of autistic traits, as a check on an autism diagnosis. We excluded from the control group anyone scoring >25, to ensure the controls were representative. This resulted in 26 individuals being excluded.

(c) The Test of Genuineness-Revised (ToG-R) [32]. This was used to validate any self-reported auditory-visual forms of grapheme-colour (GC) and sound-colour (SC) synaesthesia and was sent to all participants in order to detect true and false positives and negatives. The ToG-R is a colour chart that measures consistency in a participant’s reported colour associations to either letters (GC) or sounds (SC) over time. Participants are asked to choose from a colour chart to indicate the closest match evoked by a word (GC) or a sound (SC), and are then re-tested after an interval of at least a month, without warning. Previous work has found that synaesthetes far out-perform controls in the consistency across time in their colour reports to specific stimuli. Consistency scores were based on a point system created by Asher et al. [32] (Figure 1).

The final consistency score was calculated as a percentage:

$$\mathrm{Consistency}\;=\;\left[\frac{\sum \left(\mathrm{Points}\;\mathrm{awarded}\;\mathrm{for}\;\mathrm{items}\right)}{\mathrm{Total}\;\mathrm{number}\;\mathrm{of}\;\mathrm{items}*3}\right]*100$$

The total number of points for every item in the test was added (e.g., for the SC ToG-R, the total number of points awarded for stimuli 1–99, inclusive) and this number was divided by the total number of items with valid colour responses multiplied by three, the maximum number of points per item. This is the total number of possible points on the test (e.g., if a person gave valid answers for all 99 sounds, the total number of points possible would be 297). The total number of actual points was divided by the total number of possible points. To yield a percentage, this number was multiplied by 100.

## Results

As stated above, 172 adults with autism and 123 typical adults responded and gave electronic consent. Table 1 shows the participant characteristics.

**Table 1:** Participant characteristics

	**Number who replied**	**Number excluded**	**Final number**	**Age in years (SD)**	**% Attended university**	**% Female**	**% Right-handed**
**Autism**	172	8	164	39.52 (13.15)	60.4%	45.7%	77.6%*
**Typical**	123	26	97	41.21 (13.37)	68.0%	74.2%*	83.9%*

*P <0.05.

Eight of the adults with autism were excluded for reporting a self- but not clinician-diagnosed autism. As expected, significant differences were found for mean AQ scores between those with autism (x = 39.63, SD = 6.42) and controls (x = 16.41, SD = 5.37) [*t* [213] = 26.83, *P* <0.001]. Twenty-six of the typical adults were excluded for scoring >25 on the AQ, leaving a final non-autistic group of n = 97. No group differences were found in age or education, the latter measured by the rate of university attendance (both *P* >0.05). There was a difference in handedness and in sex ratio, in line with previous studies of autism (both *P* <0.05) [34,35]. In the autism group, n = 9 (5.5%) had high functioning autism, n = 153 (93%) had Asperger syndrome, and n = 2 (1.2%) had pervasive developmental disorder, not otherwise specified.

**Figure 2:**
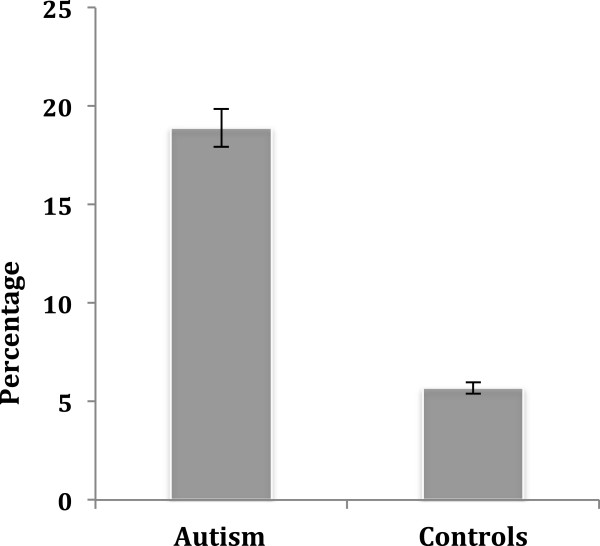
The percentage of people with synaesthesia in each group (autism vs. typical controls).

Among the 164 people with autism, 31 (14 male) met inclusion criteria for synaesthesia^b^ (a rate of synaesthesia of 18.9%), which was significantly higher than the 7.22% (n = 7 (1 male)) rate of synaesthesia among the 97 controls (χ^2^ (1, n = 261) = 6.69, *p* <0.05); 95% confidence intervals for the rate of synaesthesia in autism are ±5.99, the range for the true autism population proportion being 12.91% to 24.89%; 95% confidence intervals for the rate of synaesthesia in controls are ±5.15, the range for the true control population proportion being 2.07% to 12.37% (Figure 2). As Figure 2 shows, there is no overlap in the rates of synaesthesia in autism vs. controls. Tables 2 and 3 show an overview of what types of synaesthesia were reported in each group, and Table 4 shows the number of participants with any type of synaesthesia who completed a GC ToG-R and/or a SC ToG-R.

**Table 2:** The number who replied and the number who showed Grapheme-Colour (GC), Sound-Colour (SC), or other variants of synaesthesia, or no synaesthesia

	**n**	**Number with GC but no SC synaesthesia**	**Number with SC but no GC synaesthesia**	**Number with GC and SC synaesthesia**	**Number with other variants (not GC or SC)**	**Number (and %) reporting any type of synaesthesia**	**Number categorized as non-synaesthetes**
**Autism**	164	5	8	13	5	31 (18.9%)*	133*
**Typical**	97	2	2	1	2	7 (7.22%)	90

(*P <0.05). Statistical analyses were completed only for number and percent of participants reporting synesthesia.

**Table 3:** Number of synaesthetes with and without autism who reported different types of synaesthesia

	**Autistic synaesthetes**	**Typical synaesthetes**
**Grapheme-Colour**	18	3
**Sound-Colour**	21	3
**Taste-Colour**	7	1
**Pain-Colour**	9	1
**Smell-Colour**	5	1
**Touch-Colour**	4	0
**People-Colour**	1	0
**Taste-Shape**	1	0
**Smell-Touch**	1	1
**Sound-Touch**	1	0
**Vision-Touch**	1	0
**Sound-Taste**	1	1
**Smell–Taste**	1	0
**Movement-Sound**	1	0
**Grapheme-Personification**	3	2
**Sound-Emotion**	1	0

(Note: Cases can be in more than one cell due to co-occurrence).

**Table 4:** The number in each group who completed a GC ToG-R or SC ToG-R

	**Number who completed a GC ToG-R, of those reporting GC (but not SC) synaesthesia**	**Number who completed a SC ToG-R, of those reporting SC (but not GC) synaesthesia**	**Number who completed SC and GC ToG-R, of those reporting SC and GC synaesthesia**
**Autism**	0 /5	2 /8	1 /13
**Typical**	1 /2	1 /2	1 /1

Because the response rates were low in all cells in Table 4, no statistical analysis was conducted. Telephone follow-up to find out why ToG-Rs were not being completed revealed that participants with autism reported fatigue from the 241 possible choices. We explored if fewer colours would be less stressful [36] but participants with autism reported it would be more stressful to be unable to choose the right colour than to have to choose from a large collection of colours.

## Discussion

Our findings indicate that synaesthesia is significantly more common in adults with autism than in typical adults, based on self-report. The rate of synaesthesia in autism (18.9%) was almost three times greater than in the typical sample (7.22%), whose rate overlaps with the 4% reported previously (given confidence intervals) [1]. We predicted that synaesthesia would be more prevalent in autism than in controls if these conditions were interdependent, perhaps because they share some underlying biological causal factor, such as local neural hyper-connectivity. Four mechanisms have been proposed to account for neural hyper-connectivity: faulty axonal pruning, differences in axon guidance, disinhibition, and atypical border formation [37]. Interestingly, a recent study has revealed a significant phenotypic and genetic overlap between synaesthesia and absolute pitch [38], a trait that has also been reported to occur at increased frequency in people with autism [39,40]. This strengthens the case that autism and synaesthesia are linked at multiple levels.

It is possible that the elevated rate of synaesthesia in autism might be explained by people with autism being more likely to report abnormal sensory perceptual experiences than people without the condition. Although it is true that adults with autism score highly on sensory sensitivity questionnaires [41], we doubt this can explain the current results, because our sample included some individuals with autism (n = 3) who claimed they did not have synaesthesia, but were judged by the experimenters to have synaesthesia on the basis of their questionnaire responses. Because they reported not having synaesthesia, we conservatively considered them to be non-synaesthetes. These participants with autism declared that they did not have synaesthesia because they said they were uncertain whether their experiences counted. Thus, the high rate of synaesthesia in autism is unlikely to be an over-estimate, and could even be an under-estimate. A related possible explanation of the comorbid association might be failure in inhibition/greater cortical excitation [42]. This is in line with the high scores on sensory sensitivity questionnaires [43], and is compatible with the finding that synaesthesia occurs more frequently in autism than in the general population.

There are several limitations of this study. First, we were unable to collect complete consistency tests to validate the prevalence estimates, which will be important to explore in future work. It may be the case that traditional ToGs are not suitable for people with autism and that these will require modification. If the ToG-R is used in future studies, it should be completed in person, so that the experimenter can ensure that there is no missing data. Future studies could also consider using computerized immediate retests [44] as alternative ways for validation. It will also be interesting to test if the current results extend to children with autism, or to more impaired individuals with autism, since our sample only included high-functioning adults. Second, response rates to the initial invitations were low, which is not unusual in survey research [45], therefore, other studies must become available to confirm the observed synaesthesia prevalence rates observed, and extrapolation from the current study should be done with caution until other such surveys have been conducted. Third, this question has not yet been tested in different clinical groups to assess if this link is specific to autism. Fourth, it would be interesting to test how people with autism and synaesthesia differ from those with autism alone. As far as we know, there has not yet been a study investigating autism vs. synaesthesia vs. comorbidity between these two conditions using MRI or fMRI, which should now become a research priority in this area. Fifth, we recognize that the Synaesthesia Questionnaire is a self-report instrument that in future studies needs to be evaluated in terms of its reliability and validity. Most importantly, the next step in future research must be to explore the biological mechanisms causing the elevated rate of synaesthesia in autism.

## Conclusions

The significant increase in synaesthesia prevalence in autism suggests that the two conditions may share some common underlying mechanisms. Future research needs to develop more feasible validation methods of synaesthesia in autism.

## Endnotes

^a^Synaesthesia is a condition in which a sensation in one sensory modality automatically triggers a response in a different sensory modality. For instance, a person with ‘coloured hearing’ synaesthesia sees colours after hearing sounds… Although most synaesthetic responses are visual, synaesthesia can involve any pair of senses. Some people even experience more than one type of synaesthesia. The following are examples of what people with synaesthesia might say, “The letter q is dark brown”; “The sound of a bell is red”; “The word hello tastes like coffee”; “A toothache is shaped like a rectangle”.

^b^To be considered synaesthetic, participants had to report that they experienced synaesthesia and could not meet any of the exclusion criteria (see Methods).

## Abbreviations

AQ: Autism spectrum quotient; GC: Grapheme-colour; SC: Sound-colour; ToG-R: Test of Genuineness-Revised.

## Competing interests

The authors declare no competing interests.

## Authors’ contributions

SBC, DJ, SW, and CA designed the study. SBC and DJ drafted the manuscript and SEF, JA, and PG contributed to the manuscript. DJ and CA took responsibility for data analysis. All authors read and approved the final manuscript.
